# Neuroprotective effect of alogliptin on oxaliplatin-induced peripheral neuropathy *in vivo* and *in vitro*

**DOI:** 10.1038/s41598-020-62738-w

**Published:** 2020-04-21

**Authors:** Nao Shigematsu, Takehiro Kawashiri, Daisuke Kobayashi, Shiori Shimizu, Keisuke Mine, Shiori Hiromoto, Mayako Uchida, Nobuaki Egashira, Takao Shimazoe

**Affiliations:** 1 Department of Clinical Pharmacy and Pharmaceutical Care, Graduate School of Pharmaceutical Sciences, Kyushu University, Fukuoka, 812-8582 Japan; 2 Education and Research Center for Clinical Pharmacy, Osaka University of Pharmaceutical Sciences, Osaka, 569-1094 Japan; 3 Department of Pharmacy, Kyushu University Hospital, Fukuoka, 812-8582 Japan

**Keywords:** Neurodegeneration, Chemotherapy

## Abstract

Oxaliplatin is a platinum-based antineoplastic drug commonly used for treating colorectal, gastric, and pancreatic cancer. However, it frequently causes peripheral neuropathy as dose-limiting toxicity and is lacking a strategy for prevention. Alogliptin, a dipeptidyl peptidase 4 (DPP-4) inhibitor, is an oral antidiabetic drug. Previous studies have shown that DPP-4 inhibitors have pleiotropic effects, including neuroprotection. In this study, we investigated the effects of alogliptin on oxaliplatin-induced peripheral neuropathy using *in vitro* and *in vivo* models. In PC12 cells, alogliptin attenuated neurite disorders induced by oxaliplatin and cisplatin. The repeated injection of oxaliplatin caused mechanical allodynia and axonal degeneration of the sciatic nerve in rats. These neuropathies were ameliorated by co-administration of alogliptin. Moreover, alogliptin did not attenuate tumor cytotoxicity of oxaliplatin in the cultured colon, gastric, or pancreatic cancer cell lines and tumor-bearing mice. These findings suggest that alogliptin may be beneficial for preventing oxaliplatin-induced peripheral neuropathy.

## Introduction

Oxaliplatin is a platinum-based chemotherapeutic agent used for the standard treatment of colorectal, gastric, and pancreatic cancer. However, it has a particularly high risk of acute and chronic neuropathies. Acute neuropathy occurs in nearly all patients within hours to days after oxaliplatin infusion. Paranesthesia in the hands, feet, and the perioral region is manifested by exposure to cold temperature but is transient and reversible in most cases^1,2^.

It is believed that voltage-gated ion channels and transient receptor potential channels are involved in oxaliplatin-induced acute neurotoxicity^3–5^. Chronic neuropathy results from the repeated treatment of oxaliplatin, which is dose-limiting toxicity. Sensory and motor dysfunction occurs similar to cisplatin-induced neuropathy and may last for several months or years^2,6^. Chronic neurotoxicity is considered to be caused by morphological changes in neurons, such as axon degeneration, neuronal cell body damage, and myelin disorder^7–9^. These neuropathies remain a significant clinical problem in chemotherapy with oxaliplatin as they impact the quality of life and can lead to drug reduction or discontinuation.

Although many researches have been conducted to prevent chemotherapy-induced peripheral neuropathy, an effective treatment has yet to be found^10^. According to the clinical practice guideline established by the American Society of Clinical Oncology in 2014, no agents have yet to be recommended for the prevention of chemotherapy-induced peripheral neuropathy^11^.

Alogliptin, a dipeptidyl peptidase 4 (DPP-4) inhibitor, is clinically used for the treatment of type 2 diabetes mellitus. It lowers the blood glucose level mainly by preventing the inactivation of glucagon-like peptide-1 (GLP-1). Recently, it has received considerable attention as DPP-4 inhibitors have pleiotropic effects in addition to hypoglycemic action^12,13^. It has been reported that DPP-4 inhibitors have protective effects on central and peripheral neuropathies, including Parkinson’s disease, Alzheimer’s disease, and diabetic neuropathy^14–16^. Moreover, exenatide, a GLP-1 receptor agonist, has been shown to facilitate recovery from oxaliplatin-induced peripheral neuropathy in rats^17^. However, the protective efficacy of DPP-4 inhibitors on chemotherapy-induced peripheral neuropathy has not been previously published. In the present study, we sought to evaluate the effect of alogliptin on oxaliplatin-induced peripheral neuropathy using *in vitro* and *in vivo* models.

## Results

### Effect of alogliptin on neurodegeneration induced by chemotherapeutic agents in PC12 cells

**Figure 1:**
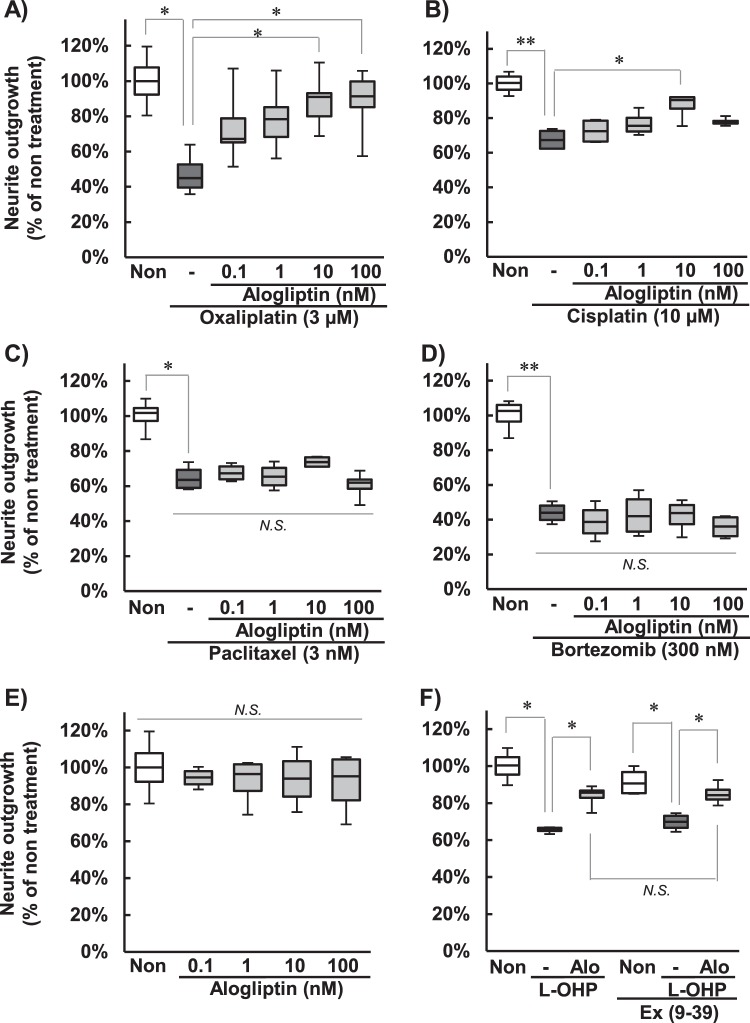
Effects of alogliptin on neurodegeneration induced by chemotherapeutic agents in PC12 cells PC12 cells were incubated with 3 µM oxaliplatin (**A**), 10 µM cisplatin (**B**), 3 nM paclitaxel (**C**), or 300 nM bortezomib (**D**) in the presence or absence of 0.1–100 nM alogliptin for 24 h. The effect of treatment with alogliptin alone was also evaluated (**E**). The figure (**F**) shows the neurite outgrowth after the co-treatment of 1 µM exendin-3 (9–39) amide (Ex (9–39)), the GLP-1 receptor antagonist, with 10 nM alogliptin (Alo) and 3 µM oxaliplatin (L-OHP) for 24 h. The neurite lengths were measured using ImageJ 1.51 software. The results are expressed as the median ± quartile values (n = 4, *P < 0.05, **P < 0.01, Games-Howel test).

In PC12 cells treated with oxaliplatin (3 µM), cisplatin (10 µM), paclitaxel (3 nM), or bortezomib (300 nM), the neurite lengths were significantly reduced (Fig. 1A–D). Co-treatment with alogliptin significantly prevented the neurite shortening induced by oxaliplatin (10 and 100 nM; *P* < 0.05; Fig. 1A) and cisplatin (10 nM; *P* < 0.01; Fig. 1B). On the other hand, the inhibition of neurite outgrowth was not improved by treatment with alogliptin in cells exposed to paclitaxel (Fig. 1C) or bortezomib (Fig. 1D). Treatment with alogliptin alone did not affect the neurite outgrowths (Fig. 1E). Exendin-3 (9–39) amide (1 µM), the GLP-1 receptor antagonist, did not reverse the neurite outgrowth by alogliptin (Fig. 1F).

### Effect of alogliptin on mechanical allodynia induced by oxaliplatin in rats

**Figure 2:**
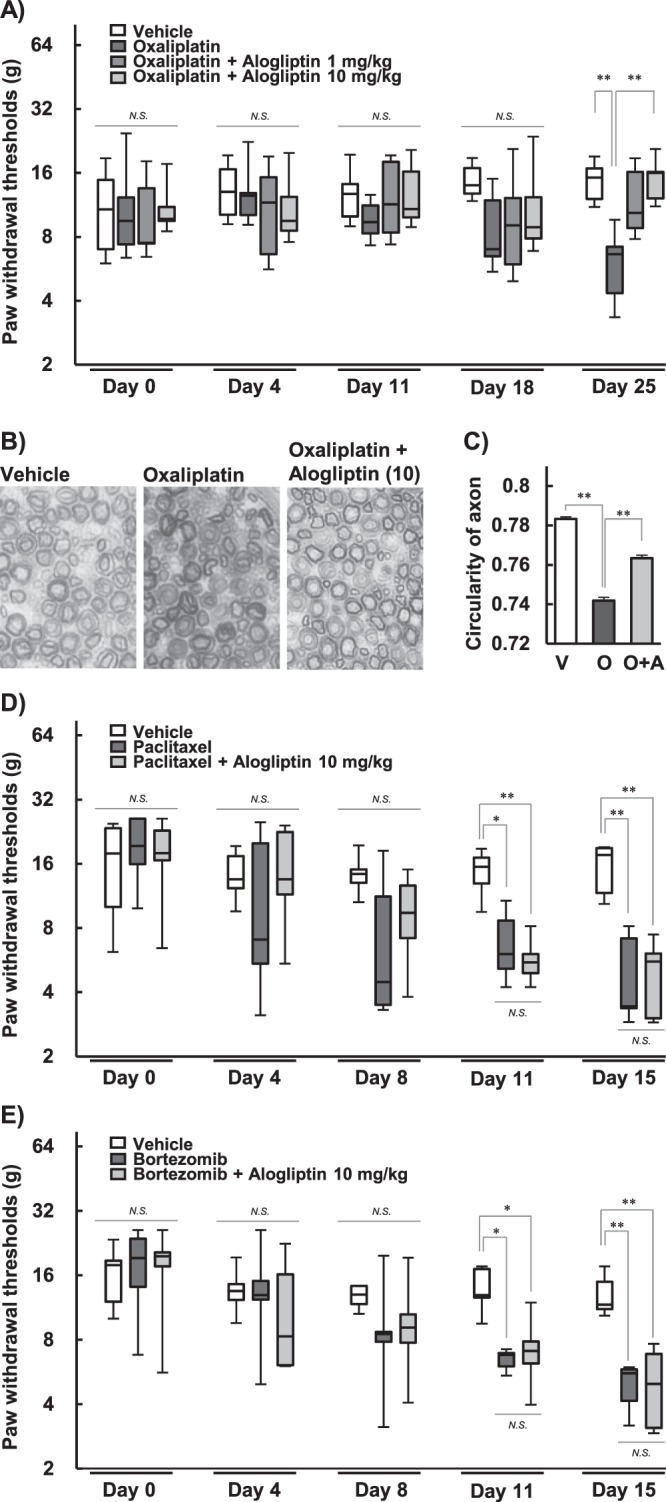
Effects of alogliptin on peripheral neuropathy induced by chemotherapeutic agents in rats Oxaliplatin (4 mg/kg) was injected intraperitoneally twice a week for four weeks. Alogliptin (1 and 10 mg/kg) was administered orally five times a week for four weeks. The von Frey test was performed before the first drug administration (day 0) and on days 4, 11, 18, and 25 (**A**). Thresholds are expressed as the median ± quartile values (n = 6–7, **P < 0.01, Games-Howel test). On day 30, sciatic nerves were harvested and stained with toluidine blue. The images (**B**) are magnified 40×. The circularity of axons (**C**) was analyzed using ImageJ 1.51 software. Circularities are expressed as the mean ± SEM. (n = 3, ^††^*P* < 0.01 compared with vehicle; **P < 0.01 compared with oxaliplatin alone, Tukey-Kramer test). Paclitaxel (4 mg/kg, i.p.) or bortezomib (0.2 mg/kg, i.p.) was treated twice a week for two weeks. Alogliptin (10 mg/kg, p.o.) was administered five times a week for two weeks. The von Frey test was performed on days 0, 4, 8, 11, and 15 (**D**,**E**). Thresholds are expressed as the median ± quartile values (n = 5–6, *P < 0.05, **P < 0.01, Games-Howel test).

Paw withdrawal thresholds significantly reduced as time progressed by the repetitive administration of oxaliplatin (4 mg/kg, i.p.) compared with the vehicle treatment on day 25 (*P* < 0.01; Fig. 2A). Co-treatment with alogliptin (10 mg/kg, p.o.) significantly improved the reduction of paw withdrawal thresholds induced by oxaliplatin on day 25 (*P* < 0.01; Fig. 2A).

### Effect of alogliptin on axonal degeneration of sciatic nerves induced by oxaliplatin in rats

In the sciatic nerves of rats, the degeneration of myelinated fibers was induced by oxaliplatin treatment (4 mg/kg, i.p.) (Fig. 2B). Repeated treatment with alogliptin (10 mg/kg, p.o.) prevented this degeneration (Fig. 2B). Oxaliplatin treatment significantly decreased the axon circularity in comparison to vehicle treatment (*P* < 0.01; Fig. 2C). Co-administration of alogliptin significantly alleviated oxaliplatin-induced reduction of the axon circularity (*P* < 0.01; Fig. 2C).

### Effects of alogliptin on mechanical allodynia induced by paclitaxel and bortezomib in rats

Paw withdrawal thresholds significantly reduced as time progressed by the repetitive administrations of paclitaxel (4 mg/kg, i.p.; Fig. 2D) and bortezomib (0.2 mg/kg, i.p.; Fig. 2E) compared with the vehicle treatment on days 11 (*P* < 0.05) and 15 (*P* < 0.01). Co-treatment with alogliptin (10 mg/kg, p.o.) improved neither paclitaxel- nor bortezomib-induced reduction of paw withdrawal thresholds (*P* < 0.01; Fig. 2D,E).

### Effect of alogliptin on oxaliplatin-induced cytotoxicity in cultured cell lines

**Figure 3:**
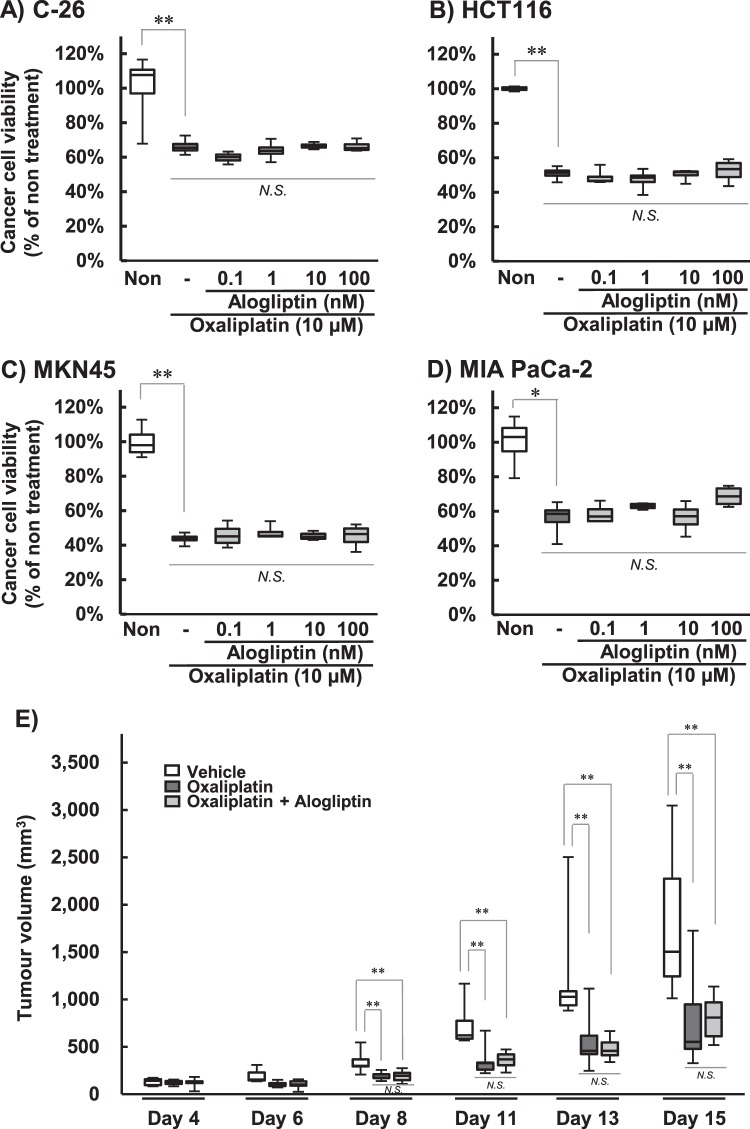
Effects of alogliptin on the antitumor activity of oxaliplatin. C-26 (**A**), HCT116 (**B**), MKN45 (**C**), and MIA PaCa-2 (**D**) cells were incubated with 10 µM oxaliplatin in the presence or absence of 0.1–100 nM alogliptin for 24 h. Cell viability was assessed using the WST-8 method. Cell viability is expressed as the median ± quartile values (n = 4, *P < 0.05, **P < 0.01, Games-Howel test). C-26 cell-implanted mice (**E**) were treated with oxaliplatin (6 mg/kg, i.p.) twice a week and alogliptin (15 mg/kg, p.o.) five times a week for two weeks. The tumor volumes were calculated as follows: volume (mm^3^) = π/6 × thickness (mm) × length (mm) × width (mm). The tumor volumes are expressed as the median ± quartile values (n = 9–10, **P < 0.01, Games-Howel test).

Exposure to oxaliplatin (10 µM) significantly reduced the cell viability in C-26, HCT116, MKN45, and MIA PaCa-2 cells (*P* < 0.01; Fig. 3A–D). Co-treatment with alogliptin (0.1–100 nM) did not affect the cytotoxicity of oxaliplatin (Fig. 3A–D).

### Effect of alogliptin on the antitumor activity of oxaliplatin in tumor cell-implanted mice

The oxaliplatin treatment (6 mg/kg, i.p.) significantly inhibited the increase in tumor volumes compared with the vehicle treatment (*P* < 0.01; Fig. 3E). Co-treatment with alogliptin (15 mg/kg, p.o.) did not affect the antitumor activity of oxaliplatin (Fig. 3E).

## Discussion

It is conceivable that neurodegeneration, such as axonopathy, neuronopathy, or myelinopathy, are responsible for oxaliplatin-dependent chronic neuropathy^7–9^. In the present study, we examined the protective property of alogliptin on oxaliplatin-induced peripheral neuropathy focused on axonopathy. In the cultured cell model, neurite outgrowth was analyzed as an index of axonopathy. Treatment with oxaliplatin, cisplatin, paclitaxel, or bortezomib shortened neurite outgrowth of PC12 cells. Alogliptin prevented only oxaliplatin- and cisplatin-induced neurite shortening. Consequently, alogliptin may exert a neuroprotective effect selectively for platinum drugs.

We also evaluated neurotoxicity using a behavioral and histological approach with the peripheral neuropathy model rats. In the von Frey test, oxaliplatin, paclitaxel and bortezomib caused mechanical allodynia. Co-administration of alogliptin inhibited the behavioral alteration induced by oxaliplatin: meanwhile alogliptin improved neither paclitaxel- nor bortezomib-induced behavioral alteration. In the histological study, axonal degeneration of the sciatic nerve was induced by repeated infusion of oxaliplatin. This means axonopathy and is in line with our *in vitro* results.

Our previous reports indicated an association between mechanical allodynia and the degeneration of axons^18,19^. Axonal degeneration induced by oxaliplatin was alleviated by co-treatment with alogliptin. These results indicate that alogliptin ameliorates oxaliplatin-induced chronic peripheral neuropathy by inhibiting neurodegeneration. Furthermore, the influence on the anticancer activity of oxaliplatin was assessed, and no impeding was observed in the cultured carcinoma cells or tumor cell-implanted mice. Hence, alogliptin is unlikely to weaken the antineoplastic efficacy of oxaliplatin.

It remains to be seen how alogliptin prevents oxaliplatin-induced peripheral neuropathy. DPP-4 inhibitors exert their activity principally by increasing the level of GLP-1. Vildagliptin suppressed the development of diabetic neuropathy by activation of GLP-1 signals^20^. Exenatide, a GLP-1 receptor agonist, inhibited oxaliplatin-induced neurodegeneration in cultured cells and rats^17^. Thus, we do not rule out the possibility that GLP-1 contributes to the antineuropathic effect of alogliptin. Conversely, GLP-1 receptor antagonist did not reverse the neuroprotective effect of alogliptin in our examination. Thus, it is thought that not only GLP-1 but also other mechanisms play a role in the neuroprotective effect of alogliptin on oxaliplatin-induced neuropathy. Many reports have shown that DPP-4 inhibitors have effects independent of GLP-1. DPP-4 is a serine exopeptidase that cleaves X-proline dipeptides from the N-terminus of polypeptides^21^. This enzyme is expressed ubiquitously and has various substrates, such as pituitary adenylate cyclase activating polypeptide (PACAP) and neuropeptide Y, in addition to GLP-1^22^. Some studies have reported these peptides have neuroprotective effects on the neurotoxicity of platinum in cultured cells and animals^23,24^. Moreover, DPP-4 inhibitors have also been reported to have antioxidant effects^25,26^. Many studies have shown that oxidative stress plays a role in oxaliplatin-induced neuropathy^27–29^. Taken together, the neuroprotective peptides and antioxidant effect, aside from GLP-1, might play a role in the neuroprotective effect of alogliptin on oxaliplatin-induced neuropathy. Interestingly, in our previous study, oxaliplatin and cisplatin, but not paclitaxel and bortezomib, down-regulated superoxide dismutase (SOD) activities in PC12 cells^30^, which means that the oxidative stress plays major roles in the neurotoxicity caused by platinum rather than by the other anticancer drugs. This mention might be a reason why alogliptin showed the neuroprotective effects specific to platinum.

Our related facilities have reported many drugs that improve oxaliplatin-induced peripheral neuropathy in the rat model^5,18,30–34^. In those reports, goshajinkigan, ifenprodil, and trifluoperazine have transient analgesic effects^31–33^, and calcium channel blockers attenuate the incidence of cold hyperalgesia in the acute neuropathy^5^. In contrast, riluzole, dimethyl fumarate, donepezil, and alogliptin, have the prophylactic effect on oxaliplatin-induced mechanical allodynia in the chronic neuropathy^18,30,34^, which is a dose-limiting toxicity in clinical. Moreover, DPP-4 inhibitors have a lower incidence of adverse events, including hypoglycemia. Therefore, DPP-4 inhibitors could be a novel preventive option for oxaliplatin-induced chronic peripheral neuropathy in patients with type 2 diabetes.

The present study demonstrates that the repeated administration of alogliptin ameliorated oxaliplatin-induced peripheral neuropathy without inhibiting antitumor efficacy in cultured cells and rodents. Therefore, alogliptin can be a preventive option for oxaliplatin-induced peripheral neuropathy.

## Methods

### Animals

We used male Sprague-Dawley rats (six-week-old, 200–250 g, Japan SLC, Inc., Shizuoka, Japan) for the peripheral neuropathy model, and BALB/c mice (six-week-old, 15–25 g, Japan SLC, Inc.) for the tumor-bearing model. Animals were bred in groups of 4–5 per cage, with a 12:12-h light-dark cycle. Animals were fed water and food *ad libitum*. All animal experiments were approved by the Experimental Animal Care and Use Committee of Kyushu University, and conducted according to the guidelines of National Institutes of Health and International Association for the Study of Pain^35^.

### Cell culture

PC12 cells (ATCC, Gaithersburg, MD, USA) were grown in Roswell Park Memorial Institute (RPMI) 1640 medium (Sigma-Aldrich Co. LLC., St. Louis, MO, USA) containing L-glutamine (2 mM), horse serum (10%), and fetal bovine serum (5%). Murine colon adenocarcinoma (C-26, Cell Resource Center for Biomedical Research, Tohoku University, Miyagi, Japan), human gastric carcinoma (MKN45, Riken, Saitama, Japan), and human pancreatic carcinoma (MIA PaCa-2, National Kyushu Cancer Center, Fukuoka, Japan) were grown in RPMI 1640 medium containing L-glutamine (2 mM) and fetal bovine serum (10%). Human colon carcinoma (HCT116, Riken) were grown in Dulbecco’s modified Eagle’s medium (DMEM, Gibco; Thermo Fisher Scientific Inc., Waltham, MA, USA) containing L-glutamine (2 mM) and fetal bovine serum (10%). Cells were cultured in humidified air supplemented with 5% CO_2_ at 37 °C.

### Drugs

In the oxaliplatin-induced peripheral neuropathy model, oxaliplatin (4 mg/kg, Yakult Honsha Co., Ltd., Tokyo, Japan) or a vehicle (5% glucose solution) was injected intraperitoneally (i.p.) twice a week for four weeks (days 1, 2, 8, 9, 15, 16, 22, and 23). Paclitaxel (4 mg/kg, Sawai Pharmaceutical Co. Ltd., Osaka, Japan) or a vehicle (50% ethanol/50% Cremophor EL) was administered intraperitoneally (i.p.) twice a week for two weeks (days 1, 4, 8, and 11) for paclitaxel-induce neuropathy model. Bortezomib (0.2 mg/kg, i.p., Janssen Pharmaceutical K.K., Tokyo, Japan) or a vehicle (saline) was treated twice a week for two weeks (days 1, 4, 8, and 11) in bortezomib model. Alogliptin (1 and 10 mg/kg, Sigma-Aldrich Co. LLC.) or a vehicle (distilled water) was administered orally (p.o.), five times a week for four weeks (days 1–5, 8–12, 15–19, and 22–26). Each drug was administered at a volume of 1 mL/kg. The doses of these drugs were determined based on previous reports^30,34,36–38^.

### Assessment of PC12 neurite outgrowths

PC12, a rat pheochromocytoma cell line, differentiates in the presence of forskolin and is used as a model of neurodegeneration. PC12 cells were seeded in collagen-coated 24-well plates (Thermo Fisher Scientific Inc.) at a density of 5.0 × 10^3^ cells/well (non-treated with chemotherapeutic agents) or 2.0 × 10^4^ cells/well (treated with chemotherapeutic agents). Neurite outgrowth was induced by 10 µM forskolin (Wako Pure Chemical Industries, Ltd., Osaka, Japan) 24 h before exposure to drugs. The cells were treated with oxaliplatin (3 µM), cisplatin (10 µM), paclitaxel (3 nM), or bortezomib (300 nM) in the presence/absence of alogliptin (0.1–100 nM) or exendin-3 (9–39) amide (1 µM), the GLP-1 receptor antagonist, for 24 h. The concentrations of these drugs were determined based on previous reports^30^. The living cells, which were not stained with trypan blue (Thermo Fisher Scientific Inc.), were analyzed using a phase-contrast microscope (CKX41; Olympus Co., Tokyo, Japan) after incubation. The neurite lengths were measured by ImageJ 1.51 software (Wayne Rasband, National Institutes of Health, MD, USA). Each experiment was performed in four wells per group.

### Von Frey test for mechanical allodynia

The von Frey test was conducted to assess the effect of alogliptin on mechanical allodynia. The test was performed on days 0 (pre), 4, 11, 18, and 25 for oxaliplatin model, on days 0, 4, 8, 11, and 15 for paclitaxel and bortezomib models. Each test was conducted before drug administration. Each rat was adapted in a wire mesh box for 30 min before the test. The method of von Frey test was described in a previous report^18^. The plantar withdrawal thresholds for stimulations by von Frey filaments (Aesthesio; DanMic Global. LLC., San Jose, CA, USA) were determined using a modified up-down method. Each experiment was performed in five to seven animals per group.

### Assessment of sciatic nerve axonal degeneration

Sciatic nerves were harvested from three rats per group anesthetized with sevoflurane (FUJIFILM Wako Pure Chemical Corporation, Osaka, Japan) on day 30. Sciatic nerves were embedded in Epon after fixing in glutaraldehyde (2%), followed by sucrose (8%) substitution. The slice samples were stained with toluidine blue^18^. Each section was evaluated using light microscopy (CKX41SF; Olympus Co.). The axon circularity was calculated as a quantitative index of axonal degeneration^37^. The quantitative data were acquired from 4,061–4,919 fibers of three animals per group.

### Tumor cytotoxicity assay in cultured cell lines

C-26, HCT116, MKN45, and MIA PaCa-2 cells were seeded in collagen-coated 24-well plates (Thermo Fisher Scientific Inc.) at a density of 2.0 × 10^4^ cells/well. On the following day, cells were treated with 10 µM oxaliplatin and 0.1–100 nM alogliptin for 24 h. Cell viability was assessed using the WST-8 method (Cell Counting Kit-8; Dojindo Laboratories, Kumamoto, Japan). Each experiment was performed in four wells per group.

### Assessment of tumor growth in tumor-bearing mice

C-26 cells (1.5 × 10^6^ cells) were subcutaneously implanted in the right paw of BALB/c mice. We selected C26 cell line-bearing mice model because it had been used in some previous studies^19,34^ and it does not require nude mice. Three days after the implantation of the tumor cells, the drug administration began. The mice were treated with oxaliplatin (6 mg/kg, i.p., on days 1, 2, 8, and 9) and alogliptin (15 mg/kg, p.o., on days 1–5 and 8–12). The dose of oxaliplatin was determined based on previous reports^19,26^. The measurements of tumor volumes were performed 4, 6, 8, 11, 13, and 15. We calculated the tumor volume as follows: volume (mm^3^) = π/6 × thickness (mm) × length (mm) × width (mm). Each experiment was performed in nine to ten animals per group.

### Sample sizes

Each sample size was calculated by the following equation: n = 2σ^2^/δ^2^ × (Z_1-α/2_ + Z_1-β_)^2^ (n, minimum sample size; α, the probability of type I error, 5%; β, the probability of type II error, 20%; σ, variance; δ, the difference between two groups). The minimum sample size in the von Frey test was calculated as five, using σ and δ values resulting from the preliminary experiments (σ = 4.0 g, δ = 7.5 g). In the same way, the minimum sample sizes of experiments of cultured cells and tumor-bearing mice were determined as four and nine, respectively (cultured cells, σ = 30.0%, δ = 15.0%; tumor-bearing mice, σ = 750 mm^3^, δ = 550 mm^3^).

### Statistical analysis

The results are expressed as the median ± quartile values or the mean ± standard error of the mean. Statistical analyzes were performed using a one-way analysis of variance followed by the Games-Howel test or the Tukey-Kramer test (Statview; Abacus Concepts, Berkeley, CA, USA) for the experiments whose sample sizes were less than 30 or more than 30, respectively. A probability level of *P* < 0.05 was accepted as statistically significant.

## Data Availability

The data that support the findings of this study are available from the corresponding author upon reasonable request.
